# Maresin 1 and CHI3L1 Levels Exhibit Opposing Trends and Correlations with Renal Dysfunction in Diabetic Nephropathy

**DOI:** 10.3390/medicina61071247

**Published:** 2025-07-10

**Authors:** Aykut Bulu, Erhan Onalan, Burkay Yakar, Gulay Bulu, Senanur Onalan Yıldırım, Mehmet Ferit Gursu, Ugur Kaplankaya, Emir Donder, Tugce Kaymaz

**Affiliations:** 1 Department of Internal Medicine, Fethi Sekin City Hospital, 23280 Elazig, Turkey; dr.aykutbulu@hotmail.com; 2 Department of Internal Medicine, Firat University, 23119 Elazig, Turkey; drugurkaplankaya23@gmail.com (U.K.); e.donder33@hotmail.com (E.D.); 3 Department of Family Medicine, Firat University, 23119 Elazig, Turkey; byakar@firat.edu.tr; 4 Department of Gynecology, Fethi Sekin City Hospital, 23280 Elazig, Turkey; gulay.figen@hotmail.com; 5 Department of Emergency, Elazıg Medikal Hospital, 23040 Elazig, Turkey; senanuronalan8@gmail.com; 6 Department of Biochemıstry, Firat University, 23119 Elazig, Turkey; fegursu@yahoo.com; 7 Department of Molecular Biology and Genetics, Faculty of Arts and Sciences, Nevsehir Hacı Bektas Veli University, 50100 Nevsehir, Turkey; tugcekaymaz@nevsehir.edu.tr

**Keywords:** MaR1, CHI3L1, diabetic nephropathy, inflammation, T2DM, biomarker

## Abstract

*Background and Objectives*: This study aimed to investigate the relationship between Maresin-1 (MaR1), Chitinase-3-like protein 1 (CHI3L1), and inflammatory as well as hematological markers in patients with type 2 diabetes mellitus (T2DM) and diabetic nephropathy (DN). *Materials and Methods*: This cross-sectional study included 90 participants divided into three groups: healthy controls (*n* = 30), patients with T2DM (*n* = 30), and patients with diabetic nephropathy (*n* = 30). The serum levels of MaR1 and CHI3L1 were measured using ELISA. Biochemical and hematological parameters were also assessed. Statistical comparisons were conducted using non-parametric tests, and correlations were analyzed via Spearman correlation. *Results*: Serum MaR1 levels were significantly higher in DN patients compared to both T2DM patients and controls (*p* < 0.01), while CHI3L1 levels were significantly lower in the DN group compared to controls (*p* = 0.007). MaR1 showed a positive correlation with CRP, BUN, and creatinine, and a negative correlation with GFR. CHI3L1 levels were positively correlated with GFR and negatively with BUN. Inflammatory markers such as CRP were elevated in the diabetic groups, while no significant differences were found in NLR values. *Conclusions*: Elevated MaR1 levels in DN patients and their correlation with renal dysfunction markers suggest that MaR1 may serve as a potential prognostic biomarker in diabetic nephropathy. The unexpected decrease in CHI3L1 levels in DN patients indicates the need for further research to clarify their role. These findings indicated that MaR1 and CHI3L1 should be further investigated in future studies as indicators for the early detection and monitoring of diabetic complications.

## 1. Introduction

Diabetes mellitus (DM) is a complex metabolic disorder characterized by impaired insulin secretion and/or action, leading to chronic hyperglycemia. Prolonged hyperglycemia results in microvascular and macrovascular complications that cause organ damage. Diabetic nephropathy (DN), one such complication, is among the most common causes of end-stage renal disease, particularly in individuals with type 2 diabetes mellitus (T2DM).

The pathogenesis of DN involves key mechanisms such as metabolic stress, oxidative damage, inflammation, and fibrotic processes. In this context, Maresin 1 (MaR1), a lipid-derived pro-resolving mediator involved in the resolution of inflammation, and Chitinase-3-like protein 1 (CHI3L1), a glycoprotein associated with immune response, tissue remodeling, and fibrosis, have become focal points in research related to diabetes and its complications.

Neutrophil–lymphocyte ratio (NLR) and platelet–lymphocyte ratio (PLR) are parameters that can be easily calculated from a practical and readily available complete blood count and have been associated with numerous medical conditions and pathologies. Studies have reported correlations between these ratios and indices and metabolic/endocrinologic disorders. Inflammatory processes play an important role in many chronic diseases, including cardiovascular disease, cancer, chronic kidney disease, and diabetes mellitus. Previous studies have shown that the neutrophil-to-lymphocyte ratio (NLR) is a systemic marker of inflammation and may have an important role in predicting short- and long-term mortality in cardiovascular disease and prognosis in patients with diabetic microvascular complications [1,2,3].

Maresin-1 was defined in human macrophages and was shown to be a lipid mediator that was active in the process of inflammatory resolution. In inflammatory states, human macrophages have been shown to produce maresin-1 via lipoxylation and enzymatic hydrolysis of docosahexaenoic acid. Inflammation and fibrosis are important pathologies in diabetes and diabetic nephropathy. Therefore, an increase in MaR-1 due to inflammation in DM and diabetic complications is an expected finding. The role of inflammation in the development of type 2 diabetes mellitus and its complications has received increasing attention because the incidence is currently increasing in the world. IL-1β, IL-6, and CRP are considered to be prognostic factors in diabetes, and anti-inflammatory lipid mediators such as maresin-1 should prevent the worsening of diabetic complications. In a rat model of diabetic nephropathy, maresin-1 exerted a protective effect on glomerular mesangial cells by decreasing the expression of ROS, NLPR3, caspase-1, and IL-1β, which are responsible for the development of diabetic nephropathy [4,5].

Conversely, CHI3L1 is a glycoprotein secreted by various cell types that structurally resembles chitinases but lacks enzymatic activity. CHI3L1 is known to regulate inflammatory responses and play a role in cell proliferation and tissue fibrosis. Recent findings indicate elevated plasma levels of CHI3L1 in diabetic patients, which may correlate with the progression of diabetic nephropathy. Additionally, CHI3L1 may contribute to DN development through m6A methylation-associated gene expression changes and M1 macrophage infiltration [6,7].

CHI3L1 has been extensively studied in the context of diabetes and its complications, with evidence supporting its involvement in inflammation, tissue remodeling, and fibrosis. Elevated serum levels of CHI3L1 are considered a potential biomarker for the diagnosis and prognosis of diabetic complications [8]. These data suggest that CHI3L1 plays a significant role in the pathogenesis of diabetic nephropathy and may influence disease progression.

Understanding the roles of MaR1 and CHI3L1 in the inflammatory and fibrotic processes associated with diabetes and diabetic nephropathy may facilitate the identification of novel targets for early diagnosis and therapeutic intervention.

MaR1 and CHI3L1 represent two critical molecules that exhibit opposing effects in the inflammation and fibrosis underlying diabetic nephropathy. MaR1 exerts protective anti-inflammatory and anti-fibrotic effects, while increased CHI3L1 expression is linked to disease progression. Therefore, we hypothesized that in diabetic nephropathy, levels of the pro-inflammatory/fibrotic marker CHI3L1 would be elevated, while levels of the pro-resolving mediator MaR1 would be altered as part of a compensatory response. The aim of this study was to investigate the serum levels of MaR1 and CHI3L1 and their relationship with inflammatory parameters such as NLR and PLR in patients with T2DM and diabetic nephropathy.

## 2. Materials and Methods

This cross-sectional study was carried out between May and June 2025 among diabetic patients monitored in the internal medicine outpatient clinic of a tertiary care center. The study was designed in accordance with the Declaration of Helsinki. The ethics committee approval was obtained from the Ethics Committee of Firat University (Ethics committee approval date: 24 April 2025, number: 2025/06-29). All of the patients were informed about the study, and their informed consent was obtained. The study sample included individuals diagnosed with type 2 diabetes mellitus (T2DM) and diabetic nephropathy who were being followed in the clinic. The control group consisted of healthy individuals who visited the same clinic for various reasons and met the eligibility criteria. The required sample size was calculated using the Web-Based Sample Size & Power Analysis Software (Malatya, Turkiye, available online: http://biostatapps.inonu.edu.tr/WSSPAS/, accessed on 1 March 2025). Based on a Kruskal–Wallis test, with a significance level (α) of 0.05, a power (1-β) of 0.80, and an effect size of 0.36, the minimum sample size needed was determined to be 26 participants per group (a total of 78). The study ultimately included 30 healthy controls (Group 1), 30 T2DM patients without nephropathy (Group 2), and 30 T2DM patients with diabetic nephropathy (Group 3), all of whom met the inclusion criteria. Participants were randomly selected from individuals presenting to the internal medicine department. The healthy control group had to be above the age of eighteen, volunteer to take part in the study, and being free of known chronic or metabolic diseases in order to be included. The study excluded participants who were obese, using hormone medication, had a known metabolic or chronic condition, had an acute infection, or used drugs.

Inclusion criteria for diabetic nephropathy and T2DM patient groups were being over 18 years of age, registered with a diabetes clinic for at least 1 year, participating in the study voluntarily, and being under follow-up in our internal medicine clinic with a previous diagnosis of T2DM and diabetic nephropathy. Those with acute infection; type 1 diabetes mellitus; pregnancy and breastfeeding; other known metabolic or chronic diseases; receiving hormonal treatment; obese; and suffering from autoimmune disease, chronic organ failure, and the use of drugs other than for DM were excluded from the study.

The study population was chosen at random from among the patients who were monitored in our internal medicine clinic. Following the American Diabetes Association’s (ADA) criteria, an internal medicine specialist made the diagnosis of diabetes mellitus. If any of the following occurred, the diagnosis was confirmed in accordance with the ADA criteria: (i) plasma glucose ≥ 126 mg/dL (7.0 mmol/L) following at least 8 h of fasting; (ii) plasma glucose ≥ 200 mg/dL (11.1 mmol/L) at 2 h during a 75 g oral glucose tolerance test (OGTT); (iii) HbA1c ≥ 6.5% (48 mmol/mol); or (iv) a random plasma glucose ≥ 200 mg/dL (11.1 mmol/L) in patients exhibiting typical hyperglycemic crisis or hyperglycemia symptoms [9]. Urine albumin creatinine ratio and estimated glomerular filtration rate were used to determine the severity of diabetic nephropathy.

The biochemical and hematological parameters of the participants were analyzed in the biochemistry laboratory of a tertiary university hospital as part of routine examination and testing. Blood urea nitrogen (BUN), creatinine, aspartate aminotransferase (AST), alanine aminotransferase (ALT), total cholesterol, triglycerides (TG), high-density lipoprotein cholesterol (HDLc), calcium, phosphorus, parathyroid hormone, C-reactive protein (CRP), plasma glucose, HbA1c, and complete blood count (CBC) were among the biochemical tests performed on each participant. High-performance liquid chromatography (HPLC) was used to assess the HbA1c levels (Adams HA-8160, BIODPC, Kyoto, Japan). In order to estimate LDLc, the Friedewald formula was utilized: total cholesterol minus HDLc minus (TG/5) equals LDLc. The SIEMENS BN II system was used to measure CRP using a nephelometric technique. Hemogram examination yielded hematological data, including “white blood cell, hemoglobin, neutrophil, lymphocyte, and platelet.” The definitions of platelet–lymphocyte ratio (PLR) and neutrophil–lymphocyte ratio (NLR) were “platelet count/lymphocyte count” and “neutrophil count/lymphocyte count,” respectively.

Blood samples taken for MaR1 and CHI3L1 values were centrifuged at 4000 rpm for 5 min. The blood samples were stored at −80 °C until the day of analysis. MaR-1 was measured using the Enzyme-Linked Immunosorbent Assay (ELISA) (SunRed, Biological Technology Co., Shanghai, China). Plasma CHI3L1 levels were measured using an ELISA kit (MICROVUE CHI3L1 EIA Kit; Quidel Corporation, San Diego, CA, USA). Absorbances were read spectrophotometrically at 450 nm in the ChroMate Microplate Reader P4300 (Awareness Technology Instruments, Palm City, FL, USA) ELISA reader. Bio-TEK ELX50 (BioTek Instruments, Winooski, VT, USA) was used as an automatic washer for washing the plates. While the intra-assay coefficient of variation (CV) of the kits was <10%, the inter-assay CV was <15%.

The SPSS 22 (Statistical Package for Social Sciences) program was used for data analysis. The Shapiro–Wilk test was used to examine whether continuous data showed normal distribution. Descriptive statistics of the data were presented as frequency (*n*) and percentage (%) for categorical data, and [median (min-max)] for continuous data because they did not show normal distribution. The Pearson Chi-square test was used in the comparison of categorical data. The Kruskal–Wallis test was used to compare more than two independent groups in continuous data that did not conform to a normal distribution. The post hoc Bonferroni test was used in pairwise comparisons between groups. Spearman correlation analysis was used to analyze the relationship between two continuous variables. The significance level was determined as *p* < 0.05.

## 3. Results

The study included 30 T2DM patients without diabetic nephropathy, 30 patients with diabetic nephropathy, and 30 healthy controls. There was no statistically significant difference between the genders of the participants (*p* = 0.107). The median age of the T2DM and diabetic nephropathy patient groups was higher than the control group (*p* < 0.001). The median values of LDL, triglyceride, glucose, and HbA1c were higher in the patient groups than in the control group. Total cholesterol levels in patients with T2DM and diabetic nephropathy were higher than in the control group. The urea and creatinine levels of patients with diabetic nephropathy were higher than both the control and T2DM patients (Table 1).

The WBC median value of diabetic nephropathy and T2DM patients was higher than the control group (*p* = 0.003). There was no statistically significant difference between the NLR levels of the groups (*p* = 0.607). The PLR level of the control group was higher than that of both the T2DM and diabetic nephropathy patients (*p* = 0.004). The CRP level of the control group was lower than that of the patients with T2DM and diabetic nephropathy (*p* = 0.002) (Table 2).

The serum MaR1 levels of patients with diabetic nephropathy were significantly higher than those of both the patients with T2DM and the control group. There was no statistically significant difference between the serum MaR1 levels of the control group and the T2DM patients. The serum CHI3L1 levels of patients with diabetic nephropathy were statistically lower than the control group (Table 3).

There was a positive correlation between the MaR1 level and CRP (*r*: 0.221; *p*: 0.036), BUN (*r*: 0.282; *p*: 0.007), and creatinine (*r*: 0.365; *p*: <0.001) levels. There was a negative correlation between MaR1 level and GFR (*r*: −0.313; *p*: 0.003). There was a significant positive correlation between CHI3L1 level and GFR (*r*: 0.271; *p*: 0.010). There was a significant negative correlation between CHI3L1 level and BUN (*r*: −0.246; *p*: 0.020) (Table 4).

## 4. Discussion

In this study, we investigated the relationship between serum MaR1 and CHI3L1 levels and inflammation parameters in patients with T2DM and diabetic nephropathy. Our findings showed that MaR1 levels were significantly higher in patients with diabetic nephropathy compared to both T2DM patients and healthy subjects. However, CHI3L1 levels were significantly lower in the diabetic nephropathy group compared to the control group. MaR1 is a soluble lipid-mediated specialized pro-resolving mediator and plays a particularly important role in the resolution of inflammation [10]. The increase in this molecule in cases of kidney injury is thought to be an attempt by the body to resolve the inflammatory process [11,12]. Previous studies have reported that inflammation caused by T2DM and diabetic nephropathy increases MaR1 levels. Increased MaR1 levels have protective properties in T2DM and diabetic nephropathy [11]. Morita et al. have reported that urinary MaR1 were lower in the subjects with stage 3–4 diabetic nephropathy than the subjects with stage 1 or 2 diabetic nephropathy and in the control group [13]. In our study, MaR1 levels correlated negatively with GFR and positively with creatinine and BUN, suggesting that MaR1 may be associated with renal dysfunction. Similarly, MaR1 levels have been reported to increase in conditions such as acute kidney injury and diabetic nephropathy in the literature [14,15].

CHI3L1 is a glycoprotein involved in the pathogenesis of various inflammatory and fibrotic diseases. Previous studies have shown that CHI3L1 levels are associated with both diabetic complications and inflammatory processes [16,17]. However, in our study, CHI3L1 levels were lower in patients with diabetic nephropathy compared to the control group. Aguilera et al. reported that concentrations of CHI3L1were significantly higher in T1DM than in the controls [18]. Previous studies have shown that elevated serum CHI3L1 levels have been found in cardiovascular disease, type 1 diabetes and type 2 diabetes and several types of cancer [19,20]. The current study findings contradict the existing literature. Thomson et al. reported that there was no association between YKL-40 and measures of insulin resistance or insulin sensitivity, indicating that YKL-40 is not directly involved in the basic pathophysiological features of T2DM [21]. Several studies have shown that variations in the YKL-40 encoding gene, CHI3L1, influence the circulating levels of YKL-40, and functional variants have been identified. Thus, the level of circulating YKL-40 is influenced by genetic factors [22,23]. This may be due to heterogeneity in the patient group, disease stage, or treatments used. Furthermore, the positive correlation of CHI3L1 levels with GFR and the negative correlation with BUN suggest that this protein may be active in the chronic phase of inflammation rather than the early phase [24,25].

In terms of inflammation markers, CRP levels were significantly higher in both the T2DM and diabetic nephropathy groups. This supports that systemic inflammation is increased in diabetic patients [26,27,28]. In addition, the positive correlation of MaR1 levels with CRP suggests that MaR1 may increase in response to inflammation. The fact that there was no significant difference in NLR levels between the groups indicates that neutrophil and lymphocyte changes may be limited in these patient groups. However, it is noteworthy that the PLR level was higher in the control group. A previous study showed that PLR was significantly higher in type 2 diabetic patients compared to healthy subjects and there was a positive correlation between HbA1c and PLR in diabetic subjects. Elevated PLR in patients with type 2 diabetes mellitus may be a reflection of the underlying inflammatory burden of the disease [29]. As HbA1c worsens due to poor diabetes control, the underlying chronic low-grade inflammatory status intensifies and thus, inflammatory markers, including PLR, increase. An increase in platelet count in diabetic patients may be another reason for the increase in PLR. Neergaard-Petersen et al. showed that platelet aggregation and platelet count were increased in prediabetic patients compared to nondiabetic patients. In addition, increased HbA1c levels were positively correlated with increased platelet aggregation and count [30]. The current study findings are consistent with data from the existing literature. Nevertheless, we recommend that the increase in PLR levels and the lack of difference in NLR levels in diabetic patients compared to controls be investigated.

The current study has some limitations. The relatively limited sample size may affect the generalizability of the results obtained. The duration of diabetes mellitus, glycemic control history, severity of diabetic nephropathy, and the treatments received were disregarded. This may have affected MaR1/CHI3L1 levels. We recommend that future studies take this limitation into account and ensure homogeneity in subgroups. In addition, more patient data and longitudinal studies are needed to explain the contradictory finding of low levels of CHI3L1.

## 5. Conclusions

In conclusion, the increase in MaR1 in the presence of diabetic nephropathy and its correlation with renal function parameters suggest that this biomarker may be a potential prognostic tool. Contrary to expectations, CHI3L1 levels were found to be lower, suggesting the need for further studies in this area. The potential use of these biomarkers in the early diagnosis of inflammation and kidney diseases can be evaluated more clearly in future studies.

## Figures and Tables

**Table 1:** Comparison of demographic and biochemical parameters between groups.

Variables	Control (*n* = 30)	T2DM (*n* = 30)	Diabetic Nephropathy (*n* = 30)	p*	p**
**Gender *n* (%)**					
Female	20 (66.7)	13 (43.3)	12 (40.0)	0.107	
Male	10 (33.3)	17 (56.7)	18 (60.0)		
**Age (years)**	22.50 (18.059.0)	52.00 (38.0–70.0)	60.50 (30.0–70.0)	***<0.001***	1–2: ***<0.001*** 1–3: ***<0.001*** 2–3: 0.199
**Total cholesterol (mg/dL)**	158.00 (108.0–286.0)	181.50 (120.0–261.0)	176.50 (78.0–275.0)	0.051	
**LDL (mg/dL)**	90.50 (20.0–148.0)	124.00 (50.0–212.0)	132.50 (42.0–206.0)	***<0.001***	1–2: ***<0.001*** 1–3: ***0.004*** 2–3: 0.383
**HDL-C (mg/dL)**	47.00 (25.0–81.0)	43.00 (29.0–90.0)	38.00 (11.0–87.0)	0.177	
**Triglyceride (mg/dL)**	85.00 (32.0–410.0)	168.50 (78.0–428.0)	161.00 (51.0–368.0)	***<0.001***	1–2: ***<0.001*** 1–3: ***<0.001*** 2–3: 0.537
**Glucose (mg/dL)**	86.20 (23.0–107.0)	136.00 (84.0–385.0)	131.00 (64.0–385.0)	***<0.001***	1–2: ***<0.001*** 1–3: ***<0.001*** 2–3: 0.154
**GFR**	90.00 (90.0–90.0)	90.00 (72.3–90.0)	62.90 (12.0–90.0)	***<0.001***	1–2: ***<0.001*** 1–3: ***<0.001*** 2–3: 0.063
**Calcium**	9.18 (8.74–9.90)	9.40 (8.61–10.40)	9.09 (7.41–10.13)	0.050	
**Phosphorus**	3.20 (2.80–3.80)	3.80 (2.90–5.60)	3.90 (2.10–9.70)	***<0.001***	1–2: ***0.001*** 1–3: ***<0.001*** 2–3: 0.455
**Urea (mg/dL)**	26.50 (15.0–43.0)	30.50 (20.0–53.0)	55.00 (13.0–153.0)	***<0.001***	1–2: 0.091 1–3: ***<0.001*** 2–3: ***<0.001***
**Creatinine (mg/dL)**	0.74 (0.49–1.05)	0.82 (0.37–1.10)	1.24 (0.55–3.82)	***<0.001***	1–2: 0.448 1–3: ***<0.001*** 2–3: ***<0.001***
**ALT (u/L)**	21.00 (5.0–70.0)	25.00 (9.0–186.0)	19.00 (8.0–44.0)	0.117	
**AST (u/L)**	21.00 (8.0–43.0)	21.00 (8.0–89.0)	19.00 (10.0–46.0)	0.595	
**HbA1c**	5.10 (4.50–5.80)	8.20 (5.60–15.80)	9.50 (6.50–13.30)	***<0.001***	1–2: ***<0.001*** 1–3: ***<0.001*** 2–3: 0.448
**Parathyroid hormone**	53.55 (1.2–115.0)	47.00 (19.0–97.3)	70.00 (28.9–151.6)	***0.034***	1–2: 0.498 1–3: ***0.012*** 2–3: 0.067

p*: multiple comparison; p** pairwise comparison. Statistically significant values are shown in italics and bold.

**Table 2:** Comparison of inflammatory markers between groups.

Variables	Control (*n* = 30)	T2DM (*n* = 30)	Diabetic Nephropathy (*n* = 30)	p*	p**
WBC	7.14 (3.80–10.00)	8.21 (6.22–15.36)	8.38 (4.06–12.00)	***0.003***	1–2: ***0.002*** 1–3: ***0.006*** 2–3: 0.741
NLR	1.72 (0.81–7.59)	1.68 (0.66–6.70)	2.15 (0.39–6.45)	0.607	
PLR	117.50 (85.20–236.30)	91.20 (41.40–340.00)	101.35 (46.0–246.0)	***0.004***	1–2: ***0.001*** 1–3: ***0.022*** 2–3: 0.345
CRP	3.00 (1.00–6.60)	3.89 (3.00–26.50)	4.75 (2.00–22.00)	***0.002***	1–2: ***0.003*** 1–3: ***0.001*** 2–3: 0.751

p*: multiple comparison; p** pairwise comparison. Statistically significant values are shown in italics and bold.

**Table 3:** Comparison of serum MaR1 and CHI3L1 levels between groups.

Variables	Control (*n* = 30)	T2DM (*n* = 30)	Diabetic Nephropathy (*n* = 30)	p*	p**
MaR1 (pg/mL)	494.69 (377.91–2862.11)	509.34 (263.89–900.88)	626.00 (414.53–1518.23)	***0.001***	1–2: 0.872 1–3: ***0.001*** 2–3: ***0.002***
CHI3L1 (ng/mL)	221.78 (37.38–735.07)	172.95 (74.12–2640.44)	160.45 (51.60–419.35)	***0.007***	1–2: 0.074 1–3: ***0.002*** 2–3: 0.170

p*: multiple comparison; p** pairwise comparison. Statistically significant values are shown in italics and bold.

**Table 4:** Spearman correlation analysis between variables.

		MaR1	CHI3L1	NLR	PLR	CRP	GFR	BUN	Cr
MaR1	*r*	1.000	0.136	0.043	−0.112	***0.221*** *	***−0.313*** **	***0.282*** **	***0.365*** **
	*p*		0.201	0.687	0.292	***0.036***	***0.003***	***0.007***	***<0.001***
CHI3L1	*r*	0.136	1.000	−0.103	0.015	−0.075	***0.271*** **	***−0.246*** *	−0.179
	*p*	0.201		0.335	0.886	0.485	***0.010***	***0.020***	0.091
NLR	*r*	0.043	−0.103	1.000	***0.542*** **	0.143	−0.156	0.096	0.074
	*p*	0.687	0.335		***<0.001***	0.178	0.143	0.367	0.487
PLR	*r*	−0.112	0.015	***0.542*** **	1.000	0.033	0.087	−0.070	−0.150
	*p*	0.292	0.886	***<0.001***		0.757	0.415	0.514	0.159
CRP	*r*	***0.221*** *	−0.075	0.143	0.033	1.000	***−0.245*** *	0.023	0.087
	*p*	***0.036***	0.485	0.178	0.757		***0.020***	0.828	0.416
GFR	*r*	***−0.313*** **	***0.271*** **	−0.156	0.087	***−0.245*** *	1.000	***−0.687*** **	***−0.758*** **
	*p*	***0.003***	***0.010***	0.143	0.415	***0.020***		***<0.001***	***<0.001***
BUN	*r*	***0.282*** **	***−0.246*** *	0.096	−0.070	0.023	***−0.687*** **	1.000	***0.694*** **
	*p*	***0.007***	***0.020***	0.367	0.514	0.828	***<0.001***		***<0.001***
Cr	*r*	***0.365*** **	−0.179	0.074	−0.150	0.087	***−0.758*** **	***0.694*** **	1.000
	*p*	***<0.001***	0.091	0.487	0.159	0.416	***<0.001***	***<0.001***	

* Correlation is significant at the 0.05 level (2-tailed). ** Correlation is significant at the 0.01 level (2-tailed). Statistically significant values are shown in italics and bold.

## Data Availability

The data presented in this study are available on request from the corresponding author due to privacy reasons.
